# Advancing Maternal Health with Long‐Acting Therapeutics: Priorities, Efficacy and Safety Considerations, and Emerging Technologies

**DOI:** 10.1002/cpt.70224

**Published:** 2026-02-02

**Authors:** Rachel K. Scott, Sharon Nachman, Ethel D. Weld, Rachel Daley, Shakir Atoyebi, Robert Bies, Catriona Waitt, Adeniyi Olagunju

**Affiliations:** ^1^ Women's and Children's Research Network, MedStar Health Research Institute Washington D.C USA; ^2^ Georgetown University School of Medicine Washington D.C USA; ^3^ Department of Pediatrics Renaissance School of Medicine, State University of New York at Stony Brook Stony Brook New York USA; ^4^ Division of Clinical Pharmacology, Division of Infectious Diseases, Department of Medicine Johns Hopkins University School of Medicine Baltimore Maryland USA; ^5^ Centre of Excellence for Long‐acting Therapeutics, University of Liverpool Liverpool UK; ^6^ Department of Biochemistry, Cell and Systems Biology University of Liverpool Liverpool UK; ^7^ Division of Pharmacokinetics, Pharmacodynamics and Systems Pharmacology, Department of Pharmaceutical Sciences School of Pharmacy and Pharmaceutical Sciences, Institute for Artificial Intelligence and Data Science, University at Buffalo Buffalo New York USA; ^8^ Department of Women's and Children's Health University of Liverpool Liverpool UK; ^9^ Infectious Diseases Institute, Makerere University College of Health Sciences Kampala Uganda

## Abstract

Maternal health remains a critical global concern, particularly in underserved populations and in low‐ and middle‐income countries where access to safe and effective therapeutics is limited. Despite the use of medications by most women during pregnancy, the exclusion of pregnant and lactating women from clinical trials has resulted in significant data gaps, hindering informed treatment decisions. As long‐acting therapeutics transition into mainstream treatment and prevention strategies, it is critical to ensure these disparities are neither perpetuated nor widened. This review synthesizes insights from the maternal health session of the July 2025 workshop of the Community of Practice for Long‐Acting Therapeutics in Maternal and Pediatric Health. It was convened and hosted by the University of Liverpool Centre of Excellence for Long‐Acting Therapeutics with funding from Unitaid. Key themes explored during the session include (1) regulatory initiatives, research networks, and data infrastructures that are driving systemic change in maternal health research over the past two decades; (2) important efficacy and safety considerations during pregnancy and lactation using insights from long‐acting antiretrovirals currently in clinical use; and (3) selected long‐acting drug delivery systems with potential applications in maternal health. Starting with maternal health priorities, here we included further insights regarding long‐acting injectable antipsychotics, long‐acting reversible contraceptives, and the role of in silico modeling in bridging existing gaps. Several immediately actionable recommendations are presented on advancing long‐acting therapeutics for maternal health priorities during pregnancy and lactation.

There are approximately 2.6 billion women (refers specifically to individuals assigned female at birth i.e., cisgender females in this article) of reproductive age (typically defined as 15–49 years) globally in 2025, accounting for 121 million pregnancies annually, of which 50% are unintended.^1^ Maternal health remains a pressing global concern, with an estimated 287,000 maternal deaths annually, most of which are preventable.^2^ However, research in pregnant and postpartum populations to address this gap has long been constrained by regulatory, ethical, and cultural barriers. The conditions presented here (**Table** **1**) represent the leading contributors to maternal morbidity and mortality. Managing them requires accessible, evidence‐based interventions within health systems that integrate maternal care to reduce preventable morbidity and mortality. These conditions continue to drive maternal morbidity and mortality globally, many of which emerge or intensify during pregnancy or lactation and require timely therapeutic interventions.

**Table 1:** Global maternal health priorities, their epidemiology, outcomes, and current interventions

Conditions	Epidemiology	Outcomes	Intervention
Postpartum hemorrhage (PPH)^97^	Accounts for approx. 27% of maternal deaths worldwide Occurs in 5–10% of deliveries Mortality may reach 100 per 100,000 live births in low‐income countries	Maternal morbidity and 27% of maternal deaths worldwide	Rapid administration of uterotonics (e.g., oxytocin, long‐acting carbetocin) Tranexamic acid Surgical intervention
Hypertensive disorders (pre‐eclampsia/ eclampsia)^98^	Contribute to 10–15% of maternal deaths Affect over 10% of pregnancies	50,000 maternal deaths annually 500,000 stillbirths annually	Antihypertensives Magnesium sulfate Close monitoring
Maternal sepsis^99^	19 million cases in 2021 Global incidence of 494 per 100,000 women Predominantly affects women aged 20–29	Major cause of mortality, especially in low‐resource settings	Broad‐spectrum antibiotics Fluid resuscitation Infection source control
Anemia^100^	Affects 35.5% of pregnant women globally Higher prevalence in low‐income regions	Increased risks of preterm birth Small for gestational age Maternal mortality	Oral and intravenous iron Nutritional support
Malaria in pregnancy^101^	Affects up to 36% globally Placental malaria occurs in 28% of cases	Maternal anemia Small for gestational age Preterm birth	Intermittent preventive treatment Antimalarial (some long‐acting therapies in development)
HIV/AIDS^102^	Disproportionately affects women of reproductive age	Increased risk of intrauterine growth restriction and preterm delivery Risks of vertical transmission in utero, intrapartum, and via breastfeeding	Antiretroviral therapy, including long‐acting therapies (received by >84% of pregnant women)
Mental health disorders^103^	Perinatal depression/anxiety affects up to 20% of women 25.5% experience anxiety in early pregnancy	Perinatal depression, anxiety, and psychosis	Selective serotonin reuptake inhibitors
Cardiovascular disease^104^	Causes 26.5% of pregnancy‐related deaths in the United States (approx. 600 annually) Significant contributor globally, especially in older/high‐risk mothers	Maternal morbidity and mortality	Antihypertensives Anticoagulants Lifestyle changes
Sexually transmitted infections^105^	1 million people daily; 1.1 million pregnant women in 2022	390,000 adverse pregnancy outcomes (syphilis)	Antibiotics and antiviral medications Vaccines
Endocrine and reproductive disorders^106^	Polycystic ovary syndrome (PCOS): affects 6–13% of reproductive‐aged women (highest in adolescents) Endometriosis: affects up to 10% (peak burden ages 25–29)	PCOS: associated with gestational diabetes, pre‐eclampsia, preterm birth, endometrial cancer, infertility, etc. Endometriosis: dyspareunia, chronic pelvic pain, infertility, etc.	Hormonal therapy Analgesics Surgery

This review presents lessons from regulatory initiatives and research networks that are driving systemic change in maternal health research over the last two decades. Considerations during pregnancy and lactation are discussed using insights from long‐acting antiretrovirals (LA‐ARVs), long‐acting injectable (LAI) antipsychotics, and long‐acting reversible contraceptives (LARCs) currently in clinical use. A summary of selected long‐acting drug delivery systems is provided, with emphasis on pregnancy‐ and lactation‐specific considerations. The final section explores the potential applications of pharmacokinetic modeling in advancing the field.

While specific therapeutic areas such as HIV, psychosis, and contraception have been used as examples, the considerations described have broader relevance. Similar approaches will be essential to ensure timely research and uptake of long‐acting therapeutics for other maternal health indications during pregnancy and lactation. These represent a synthesis of presentations from the maternal health session of the July 2025 workshop of the Community of Practice for Long‐acting Therapeutics for Maternal and Paediatric Health, hosted by the Centre of Excellence for Long‐Acting Therapeutics (CELT) at the University of Liverpool.

## INITIATIVES DRIVING SYSTEMIC CHANGE IN MATERNAL HEALTH RESEARCH

### Regulatory initiatives

The United States regulatory framework increasingly supports the ethical and scientific inclusion of pregnant women in clinical research. Under the Federal Food, Drug, and Cosmetic Act (FD&C Act, Section 505), the FDA can require postmarketing studies to assess drug safety in special populations, including during pregnancy and lactation.^3^ The IND safety reporting rule (21 CFR 312.32) mandates the reporting of serious adverse events such as congenital anomalies, enabling oversight of pregnancy‐related outcomes. Ethical protections are outlined in 45 CFR 46 Subpart B, which permits research involving pregnant women when prior nonclinical and nonpregnant human data exist, and when the study offers direct benefit or poses minimal risk.^4^ Complementary FDA guidance documents—including the Pregnancy and Lactation Labeling Rule (PLLR) and the 2018 guidance on scientific and ethical considerations—emphasize the importance of pregnancy‐specific pharmacokinetic studies and informed labeling.^5^, ^6^ While this improved clarity, a 2020 analysis revealed that fewer than 20% of new drug labels included human data on pregnancy or lactation, and many remained out of PLLR compliance.^7^ The European Medicines Agency is also making regulatory changes, encouraging the inclusion of pregnant and lactating individuals in clinical trials to ensure safer, more informed treatment decisions.^8^ These efforts are supported by broader European Union strategies that mandate gender‐sensitive research and promote equitable healthcare access, positioning Europe as a leader in maternal pharmacology and therapeutic innovation.^9^, ^10^ In the United States, the establishment of the Task Force on Research Specific to Pregnant Women and Lactating Women (PRGLAC) under the 21st Century Cures Act (2016) marked a pivotal shift in maternal health research. Convened by the Eunice Kennedy Shriver National Institute of Child Health and Human Development (NICHD), PRGLAC advocated for the earlier inclusion of pregnant individuals in clinical trials—ideally beginning in Phase 1—and emphasized the importance of linking maternal–infant health records and improving drug registries.^11^, ^12^ Despite these recommendations, implementation has been very limited, and regulatory progress has been incremental at best.^13^

### Research networks

Obstetrical trials face other persistent barriers: perceived limited eligible populations, high costs, and complex long‐term follow‐up requirements. These challenges reduce the return on investment for pharmaceutical companies, especially given the short duration of treatment and limited market scope. To address these gaps, several networks and initiatives have emerged over the last two decades. A few examples are highlighted below:

- The International Maternal Pediatric Adolescent AIDS Clinical Trials (IMPAACT) Network, funded by NIH since 2006, leads global research to improve outcomes in pregnancy, infancy, childhood, and adolescence among populations affected by HIV and tuberculosis.^14^ Operating across 52 sites in 12 countries, it has produced pivotal evidence shaping treatment and prevention strategies—most notably through studies like PROMISE (Promoting Maternal and Infant Survival Everywhere), which informed global guidelines on HIV treatment during pregnancy and postpartum.^15^ IMPAACT continues to innovate, expanding into LA‐ARVs. Studies such as IMPAACT 2050 are expected to fill important gaps regarding the safety and pharmacokinetics of LA‐ART in pregnant/postpartum women. These efforts exemplify how to drive equitable, evidence‐based advancements in therapeutics for women during pregnancy and postpartum.
- The Maternal Pediatric Precision in Therapeutics (MPRINT) Hub, established in September 2021 with Indiana University and Ohio State University as co‐leads, advances maternal and pediatric pharmacology through outreach, pharmacometric support, and a publicly accessible knowledge base. Funded by NICHD, MPRINT supports research across key therapeutic areas—including obstetrics, lactation, and pediatrics—with specialized cores focused on modeling, real‐world data, biospecimen access, and education. Its mission is to improve precision therapeutics for underrepresented populations, such as women during pregnancy and lactation, and children.^16^
- The European Union, through initiatives like Horizon Europe and the Innovative Medicines Initiative, is advancing research on women's health during pregnancy and lactation. Flagship projects such as ConcePTION—launched in 2019 and involving 88 organizations across 22 countries—aim to improve access to reliable data on medication safety for women during pregnancy and lactation. By building a coordinated biomedical ecosystem, these efforts address longstanding gaps in drug labeling, clinical guidance, and regulatory inclusion, helping ensure safer and more informed therapeutic decisions during these critical life stages.
- The Accelerating Innovation for Mothers project is a global initiative led by the Concept Foundation and partners, aimed at revitalizing therapeutic development for pregnancy‐specific conditions like preeclampsia and postpartum hemorrhage. It maintains a comprehensive maternal health product pipeline, develops target product profiles, and supports research networks based in low‐ and middle‐income countries (LMICs) to improve regulatory trial readiness and accelerate access to innovative maternal health solutions.
- The Maternal‐Fetal Medicine Units (MFMU) Network, established by NICHD in 1986, is a United States‐based multicenter research network focused on improving maternal and fetal outcomes. Operating across 14 university‐affiliated centers and a data coordinating hub, MFMU conducts large‐scale clinical trials and cohort studies, representing over 160,000 annual deliveries. Its research has shaped clinical practice by identifying effective interventions and discontinuing those found to be ineffective or harmful.^17^
- The Centre of Excellence for Long‐acting Therapeutics (CELT) at the University of Liverpool is a global research hub advancing long‐acting medicines through interdisciplinary collaboration in pharmacology and materials chemistry. With funding from Unitaid, CELT is developing long‐acting treatment/prevention strategies for diseases like malaria, tuberculosis, and hepatitis C, with a focus on improving access and adherence in LMICs. The Community of Practice for Long‐acting Therapeutics for Maternal and Pediatric Health, hosted by the Perinatal Pharmacology Group in CELT, brings together a globally representative group of stakeholders. The objective is to catalyze the development and availability of long‐acting therapeutics for maternal and pediatric health, particularly in LMICs.

### Data infrastructures

Large‐scale databases provide critical insights that may be overlooked in early‐phase clinical trials, supporting secondary research and enabling data harmonization across studies. Resources with applications in pregnancy and lactation research include:

- All of US Research Program—integrates electronic health records, surveys, and biospecimens to support research across diverse populations, including women during pregnancy
- FDA Adverse Event Reporting System—postmarketing surveillance data, including exposure and adverse drug events during pregnancy and lactation
- Healthcare Cost and Utilization Project—hospital care and outcomes data.
- Medical Expenditure Panel Survey—health services cost and utilization data.
- NICHD Data and Specimen Hub—repository of deidentified biospecimens and clinical data^18^
- PKRxiv—open‐access platform for sharing individual‐level pharmacokinetic data, including pregnancy‐relevant datasets (e.g., from IMPAACT studies), developed by the University of Liverpool (also in this issue)^19^
- Pregnancy Risk Assessment Monitoring System—maternal attitudes, behaviors, and experiences before, during, and shortly after pregnancy.
- Various Pregnancy Exposure Registries—observational studies that collect data on the effects of prescription medicines used during pregnancy. They are often sponsored by pharmaceutical companies, and the data help inform drug labeling and clinical guidance.

### Lessons for long‐acting therapeutics for maternal health

While challenges remain, these initiatives are driving progress in research for maternal health therapeutics. They underscore the importance of early inclusion of pregnant and postpartum women in clinical trials, sustained investment in research infrastructure, and transparent data sharing. Strategic collaboration, stakeholder engagement, and regulatory clarity have proven essential in overcoming longstanding barriers to maternal health innovation. It is vital to build on these foundations as long‐acting therapeutics emerge as promising tools to address persistent challenges in maternal health. In HIV treatment and prophylaxis, LAI antiretrovirals such as cabotegravir (CAB), rilpivirine (RPV), and lenacapavir (LEN) have demonstrated high efficacy, improved adherence, and reduced stigma. These benefits are especially critical for preventing rebound viremia and mother‐to‐child transmission during the postpartum period, when adherence to daily oral regimens often declines. Decades of use of LAI antipsychotics and LARCs have similarly shown better clinical outcomes compared with daily oral medications.

The development pipeline for long‐acting therapies in other maternal health indications remains limited. Currently, only one product is in clinical development for preeclampsia: CBP‐4888, a subcutaneously delivered siRNA therapy now in Phase I trials. This investigational agent comprises lipid‐conjugated siRNA duplex oligonucleotides (siRNA‐2283 and siRNA‐2519) that target two sFLT1 mRNA isoforms, aiming to selectively suppress preeclampsia‐associated sFLT1 expression in the placenta.^20^ Among the repurposed daily oral antimalarial drugs under early investigation as long‐acting therapies, only artemether and lumefantrine are not contraindicated in pregnancy.^21^ However, it remains too early to determine whether their micro‐array patch format will be suitable for adult use, given current limitations in drug‐loading capacity. To support the safe and effective integration of long‐acting interventions into maternal health, evidence‐based recommendations are needed—not only for antipsychotics, contraceptives, and HIV but also for upcoming long‐acting therapeutics for pre‐eclampsia, malaria, and other areas of priority. Future research and policy must be guided by lessons from previously highlighted initiatives.

Key priorities regarding the use of long‐acting therapeutics during pregnancy and lactation include the following:

- Designing trials that capture pregnancy‐ and lactation‐specific pharmacokinetics in both maternal and infant circulation, and in breastmilk.
- Understanding drug delivery systems (DDS) in the context of pregnancy and lactation.
- Use of computational modeling tools to elucidate difficult‐to‐study scenarios.
- Regulatory clarity and incentivizing the industry to prioritize evidence generation.
- Leveraging biobanks and real‐world data for long‐term maternal and infant outcomes.

These will accelerate the development and equitable delivery of long‐acting therapeutics that address critical maternal health needs, ultimately improving outcomes for mothers and their children worldwide.

## EFFICACY AND SAFETY CONSIDERATIONS FOR LONG‐ACTING THERAPEUTICS DURING PREGNANCY

Pregnancy is a unique physiologic state with notable shifts in drug absorption, distribution, metabolism, and elimination.^22^ Although exposure to oral and long‐acting medications is both (potentially) altered by these physiologic changes of pregnancy, the relevance of these pregnancy‐associated changes differs considerably by route of administration. Notably, long‐acting therapeutics obviate pharmacokinetic changes due to decreased absorption associated with nausea and vomiting of pregnancy, as well as alterations in gastric emptying, gastric pH, and first pass effect. Changes in absorption may still be relevant for injectable formulations associated with changes in fat distribution. Although inhibition and induction of gut enzymes are no longer applicable, this is not the case for changes in inhibition and induction of hepatic enzymes.

### LA‐ARVs

Currently approved LA‐ARVs include CAB, RPV, and LEN. CAB is an integrase strand transfer inhibitor used alone for HIV prevention (intramuscular injection every eight weeks), or for HIV treatment co‐administered with the non‐nucleoside reverse transcriptase inhibitor RPV (separate intramuscular injections every four or eight weeks).^23^ LEN, a capsid inhibitor, is administered subcutaneously every six months for prevention or treatment (with other ARVs).^24^ Despite the critical importance of pharmacokinetic and safety data for ARVs in pregnancy and lactation, there is a 6‐year median lag between the availability of this data in the general population and data specific to pregnancy. While robust pharmacokinetic and safety data for LA‐CAB and LA‐RPV in pregnancy and lactation will likely become available close to this 6 year mark,^25^, ^26^ LEN HIV prevention trials broke convention by taking advantage of regulatory provisions in the FDA Act highlighted previously. This allowed pregnant participants to continue and enabled the collection of contemporaneous safety data.

### LA‐CAB/RPV

#### What we know

Initial pregnancy data came from phase II/III trials where participants switched to oral ART upon pregnancy diagnosis.^27^ Tail‐phase pharmacokinetic data in seven pregnant participants remained within non‐pregnant ranges, with sustained viral suppression. Among 25 pregnancies, outcomes included six miscarriages, eight elective abortions, one ectopic pregnancy, and 10 live births—consistent with background rates. Additional data include a case report and a series of 23 pregnancies continuing LA‐CAB/RPV (**Table** **2**). The case report showed CAB and RPV concentrations above protein‐adjusted IC_90_ (PA‐IC_90_) and cord‐blood to maternal plasma (CB:MP) ratios of 1.27 for CAB and 0.6 for RPV.^28^ The case series reported viral suppression in those continuing LA‐CAB/RPV and HIV‐negative neonates at delivery, though the study did not include pharmacokinetics.^29^ CAB in HIV prevention was studied in HPTN 084, comparing LA‐CAB to daily oral emtricitabine and tenofovir disoproxil fumarate (F/TDF). In the open‐label extension, pregnant participants could continue their regimen. Preliminary results showed no increase in adverse outcomes by regimen or timing of CAB initiation (**Table** **2**). No maternal deaths or HIV infections occurred. Infant outcomes were within background rates; four infant deaths (one congenital anomaly, three respiratory distress) were not attributed to CAB.^30^ Pharmacokinetic data from 50 participants showed troughs above 4× PA‐IC_90_ across trimesters.^31^ Most of the available data on RPV pharmacokinetics during pregnancy are primarily from oral formulations. Exposures decrease by 20–50% in later trimesters due to CYP3A4 induction, but no dose adjustment is recommended. Transplacental transfer was reported with a CB:MP ratio of 0.50–0.55.^32^, ^33^, ^34^, ^35^, ^36^ The Antiretroviral Pregnancy Registry (https://www.apregistry.com/) found no increased birth defect risk with first‐trimester exposure.

**Table 2:** Summary of research to date and gaps in clinical pharmacology data for LA‐ARVs in pregnancy and lactation

Drug	LA‐ARV PK Initiation	Placental transfer	Breastmilk transfer	Safety
CAB	(+) Prior (−) During	(+) Case report CB:MP 1.27	(+) Variable transfer (postpartum initiation)	(+) PrEP (RCT) (+) Treatment (case series)
RPV	(+) Prior—case report (−) During	(+) Case report CB:MP 0.6 (LAI) (+) CB:MP 0.55 (oral)	(+) Case report	(+) Treatment (case series) (+) APR data (oral)
LEN	(+) Prior (−) During	(?)	(+)	(+) PrEP (RCT) (+) Treatment (case report)

(−) no data, (+) data exist, (?) data may exist, but have not been published/presented.

APR, antiretroviral pregnancy registry; CAB, cabotegravir; LA‐ARV, long‐acting antiretroviral; LEN, lenacapavir; PrEP, pre‐exposure prophylaxis; RCT, randomized controlled trial; RPV, rilpivirine.

#### What it means

Although pregnancy is known to alter drug metabolism through changes in hepatic enzyme activity, plasma volume, and protein binding, current data suggest that these physiological shifts may not substantially compromise CAB pharmacokinetics in the context of CAB‐LA or LA‐CAB/RPV. CAB is primarily metabolized by UGT1A1, an enzyme whose activity may be modestly increased during pregnancy, potentially enhancing CAB clearance. However, available pharmacokinetic data—including tail‐phase levels in pregnant participants and trough concentrations from HPTN 084—demonstrate drug exposures consistently above the PA‐IC_90_, supporting sustained viral suppression. These findings, alongside reassuring maternal and infant outcomes, suggest that pregnancy‐related changes in UGT1A1 activity may not necessitate dose adjustments, though further dedicated studies are warranted to confirm these observations and guide clinical practice. Based on the known impact of pregnancy on CYP3A4 activity and pregnancy pharmacokinetic data for the daily oral RPV, pregnancy is expected to affect LA‐RPV exposure. The clinical significance will likely depend on the duration of LA‐RPV and the extent of virological suppression before pregnancy.

### LEN

#### What we know

The pivotal trial data that informed LEN regulatory approval came from PURPOSE 1, a Phase III, double‐blind, randomized controlled study that evaluated the efficacy of six‐monthly subcutaneous LEN vs. daily oral emtricitabine/tenofovir alafenamide (F/TAF) or F/TDF for HIV prevention in adolescent girls and young women in South Africa and Uganda.^37^ At the time of the interim analysis, there were 510 pregnancies among 487 participants—193 in the LEN group, 219 in the F/TAF group, and 98 in the F/TDF group—with 277 delivery outcomes and 233 ongoing pregnancies (**Table** **2**). Pregnancy and birth outcomes were similar to those expected for the general population. No documented incident of HIV was reported among participants receiving LEN. Preliminary pregnancy and lactation pharmacokinetic data were presented at AIDS 2025.^38^ The study team reported on 184 participants on LEN with a total of 193 pregnancies: 88 (45.6%) ongoing and 105 (54.4%) with pregnancy/birth outcome data. Of completed 105 pregnancies, there were 52 live births (49.5%) and 53 abortions (50.5%), including 30 induced/elective abortions (28.6%), 20 spontaneous abortions (19.0%), and 3 stillbirths (2.9%). Maternal pregnancy‐associated adverse events were rare, most commonly hypertensive disorders of pregnancy (*n* = 4) and hyperemesis gravidarum (*n* = 3). No HIV infections occurred in this group. The population pharmacokinetic (popPK)analysis did not predict statistically significant differences LEN exposure by pregnancy trimester or postpartum status compared with non‐pregnant/non‐lactating women.

#### What it means

LEN is primarily eliminated via hepatic metabolism, with minimal renal clearance, and is known to be a substrate of CYP3A4 and P‐glycoprotein (P‐gp) transporters.^39^, ^40^ During pregnancy, physiological changes—including increased plasma volume, altered protein binding, and upregulation of CYP3A4 and P‐gp activity—can potentially reduce LEN exposure by enhancing clearance. However, data from PURPOSE 1 suggest that LEN exposure remains stable across pregnancy trimesters and postpartum, with trough concentrations consistently above the threshold required for viral suppression. This may be attributed to LEN's long half‐life, high potency, and depot formulation, which buffers against transient metabolic changes. Overall, these findings suggest that pregnancy‐induced physiological changes may have minimal clinical impact on LEN pharmacokinetics, although continued monitoring and dedicated studies are essential to confirm these observations and guide its use in perinatal HIV prevention strategies.

### LAI antipsychotics

#### What we know

LAI antipsychotics have multiple advantages over oral antipsychotics (OAs) and are recommended for patients at risk of recurrent relapse and hospitalization due to poor adherence. A self‐controlled case series study of people in Hong Kong with schizophrenia (*n* = 70,396) found that LAI antipsychotics were associated with a lower risk of disease relapse and hospitalization than OAs, with incidence rate ratios between 0.52 and 0.63, without an increased risk of adverse events.^41^ A greater reduction in outcome events was observed in early LAI antipsychotic initiators vs. late initiators. Hence, previously viewed as an option for a small subgroup of patients with noncompliance, frequent relapses, or who pose a risk to others, LAI antipsychotics are now considered the optimal treatment option for any patient where maintenance antipsychotic treatment is indicated.^42^

However, currently available LAI antipsychotics are not licensed for use in pregnant and breastfeeding women due to uncertainties about their safety. There is an acute lack of data on the pharmacokinetics of LAI antipsychotics during pregnancy and lactation.^43^, ^44^, ^45^ Our current thinking is guided by very limited data on daily OAs, for some of which transplacental passage has been reported.^46^ The following are based on a retrospective analysis of routine therapeutic drug monitoring data (512 sparse measurements, 110 pregnancies, 103 women) by Westin et al.^47^ regarding serum concentration during the third trimester compared with pre‐pregnancy state:

- Orally administered quetiapine: 76% decrease.
- Orally administered aripiprazole: 52% decrease.
- Clozapine and olanzapine: No significant changes observed.
- Orally administered perphenazine: 69% decrease (*n* = 7).
- Intramuscularly administered perphenazine: 57% decrease (*n* = 8).

As these and other antipsychotics are metabolized by CYP450 enzymes, there is a need for prospective assessment of their pharmacokinetics during pregnancy. Current knowledge regarding the extent and clinical consequences of in utero fetal exposure to LAI antipsychotics is equally based on very limited data on daily oral formulations:

- Cord‐to‐maternal plasma concentration ratios ranging from 0.08 to 1.91 have been reported in studies involving between 1 and 27 participants, without relevant covariates in most cases.^45^
- About 37% of the infants exposed to antipsychotics in utero had some degree of respiratory distress at birth, higher than the nationally reported rate of 28%, with 43% requiring special care nursery or neonatal intensive care unit admission with higher doses or concurrent mood stabilizer.^48^
- Compared with atypical antipsychotics, babies exposed to typical antipsychotics in utero (*n* = 147) were more likely to experience postnatal symptoms (21.6% vs. 15.6%), including respiratory, digestive, cardiac, and nervous system concerns.^49^

Some of these conclusions were based on sparse drug concentration measurements, and sometimes there is no accurate data on the time after the dose. The extent of fetal exposure associated with observed symptoms is also currently unknown. A recent physiologically‐based pharmacokinetic (PBPK) modeling study found higher fetal *C*_max_ and a higher cumulative exposure with LAI aripiprazole and LAI olanzapine compared to their daily oral formulations.^50^

#### What it means

There is a need for more robust data on the pharmacokinetics of LAI antipsychotics during pregnancy, including potential pregnancy‐induced changes in exposure across trimesters and the resulting in utero fetal exposure.

Importantly, any expectation of differences in fetal exposure between LAI and daily oral antipsychotic will likely involve steady‐state peak‐to‐trough fluctuations, which are expected to be minimal for LAI in general leading to less advert effects. However, a comparison of this across multiple antipsychotics showed that there is no general rule. Observed ratios were 1.47 (paliperidone extended‐release, once daily) to 3.30 (active‐moiety risperidone, once daily) for OAs, and 1.56 (paliperidone palmitate, once monthly) to 4.0 (olanzapine pamoate, once every four weeks).^51^

To address the evidence gap, the Long‐acting Antipsychotics for Maternal Ill‐Health During Pregnancy and Postpartum study (LAMP; NCT05766007), led by the University of Liverpool, is currently recruiting participants in clinical sites across Nigeria. It will evaluate the pharmacokinetics of popularly used LAI antipsychotics during pregnancy and postpartum, including in utero exposure assessed at delivery, breastmilk pharmacokinetics, and breastfed infant exposure.

### Breastfed infant exposure to maternal long‐acting vs. immediate‐release drugs

Despite advances in the ability to investigate lactation pharmacokinetics, together with clear guidance on study design and the ethical imperative to perform these studies,^52^ there is often less emphasis on understanding the safe, effective use of drugs in lactation compared with pregnancy.^52^, ^53^ This gap is particularly evident across therapeutic areas such as psychiatry, infectious diseases, and chronic conditions. Historically, breastfeeding women have been excluded from clinical trials due to concerns about infant safety, liability, and logistical complexity. However, this exclusion has led to fragmented guidance and inequitable access to evidence‐based care.^53^, ^54^ As long‐acting therapeutics become mainstream options in multiple therapeutic areas—including antiretrovirals, antipsychotics, and contraceptive implants—it is important to generate the evidence needed to guide their use in postpartum women. Their sustained pharmacokinetic profiles may result in continuous low‐level drug transfer into breast milk, with potential for accumulation in the infant, particularly for agents with long half‐lives, active metabolites, or high milk‐to‐plasma ratios. The implications may be amplified during early infancy, when hepatic and renal clearance mechanisms are immature.^55^, ^56^, ^57^

#### What we know

##### LA‐ARVs

There is no published work describing the lactation pharmacokinetics of LA‐ARVs used for the treatment of HIV. Some data were presented at the 13th International AIDS Society Conference on HIV Science (July 2025) in the context of HIV pre‐exposure prophylaxis (PrEP):

- LEN (PURPOSE 1): Median milk‐to‐plasma ratio: 0.52 (IQR: 0.38–0.77) in 102 matched maternal plasma and breastmilk pairs. Median infant‐to‐maternal plasma ratio: 0.02 (IQR: 0.01–0.05) in 98 matched mother–infant pairs.^38^
- LA‐CAB (Tshireletso): Median milk‐to‐plasma ratio of 0.014 (IQR: 0.011–0.019); median relative infant dose (RID) of 2.5% in 27 lactating mother‐infant pairs.^58^
- LA‐RPV: No data are available for the long‐acting formulation. However, in two mothers taking oral RPV (25 mg daily), the milk‐to‐plasma ratio was 1.08, with low but detectable infant plasma levels, consistent with other ARVs in the same class.^59^, ^60^

While these findings suggest low infant exposure, full reports are needed to assess generalizability and potential drug accumulation over time, for instance, in the context of breastfeeding at steady‐state. IMPAACT 2050 is a phase IV observational study that will evaluate real‐world pharmacokinetics in women continuing CAB/RPV‐LA during pregnancy and postpartum. The DOLPHIN3 trial (NCT05852986), funded by EDCTP and launching in 2026, will evaluate a temporary switch from oral regimens to LA‐CAB/RPV between 6 weeks and 12 months postpartum. The primary outcome is virological suppression and retention in care, with lactation pharmacokinetics as a secondary objective. These studies are notable for prioritizing breastfeeding mothers, addressing adherence challenges, and transmission risks during the postpartum period. While pharmacokinetics may not differ substantially between treatment and prevention contexts, how best to handle ongoing postpartum exposure in women who discontinue LA‐ARVs at the start of pregnancy warrants further exploration.

##### LAI antipsychotics

LAI antipsychotics are increasingly used in perinatal mental health care due to their potential to improve adherence and maintain stable plasma concentrations. Available data suggest that infant exposure to LAI antipsychotics during lactation is generally low and comparable to oral formulations.

- Risperidone LAI has a RID ranging from 2.8% to 9.1% and is rated L2 (safer) under Hale's lactation risk categories, with limited reports of sedation or extrapyramidal symptoms (EPS) in infants.^61^
- Haloperidol LAI, with an RID of up to 12%, is rated L3 (moderately safe) due to its higher D2 receptor potency and associated EPS risk.^62^
- Aripiprazole LAI shows an RID between 0.7% and 6.4%, also rated L3, reflecting limited lactation data but a favorable pharmacologic profile as a partial agonist. A systematic review advised cautious use of aripiprazole during lactation, noting low transfer into breastmilk and minimal adverse effects in infants.^63^
- Paliperidone, the active metabolite of risperidone, lacks direct RID data but is presumed to have a similar exposure and safety profile.

The LAMP study (NCT05766007) is expected to generate data on breastmilk pharmacokinetics and infant exposure to LAI antipsychotics during lactation.

### Long‐acting reversible contraceptive (LARC)

LARCs, including intrauterine devices (IUDs) and subdermal implants, are widely endorsed as safe and effective for use during lactation. This is based on available data from clinical lactation studies, which indicate very low concentration in breastmilk and negligible presence in breastfed infant plasma (**Table** **3**).

**Table 3:** Summary of breastmilk pharmacokinetics of long‐acting reversible contraceptives

Drug/route/dosing frequency	Formulation type	Milk pharmacokinetics
Levonorgestrel IUD *Intrauterine* *3–8 years*	Levonorgestrel‐releasing IUD (13.5 to 52 mg)	Shikary et al. (1987)—Median ± SD milk *C*_ss_: 46 ± 16 pg/mL; *T*_max_: 2 days; milk‐to‐plasma ratio: 0.08–0.13; Infant exposure: 30 ± 12 pg/mL; milk to infant serum transfer: 75 ± 17%.^107^
	Subdermal implant (2 × 75 mg)	Shikary et al. (1987) —milk *C*_ss_: 67 ± 27 pg/mL; *T*_max_: 2 days; milk‐to‐plasma ratio: 0.04–0.12; infant exposure: 46 ± 18 pg/ml; milk to infant serum transfer: 68 ± 20%.^107^
	Minipill (30 μg daily oral dose) —*Note: included for comparison*	Shikary et al. (1987)—milk *C*_max_: 50 ± 12 pg/mL; *T*_max_: 2 hours; milk‐to‐plasma ratio: 0.04–0.12; infant exposure: 20 ± 4 pg/mL; milk to infant serum transfer: 32 ± 3%.^107^
Etonogestrel implant *Subdermal* *Up to 3 years*	Implant (68 mg)	Reinprayoon et al. (2000) —milk *C*_max_: ~0.02–0.05 ng/mL; *T*_max_: 2–4 weeks; milk‐to‐plasma ratio: 0.05–0.1; estimated infant exposure: 0.002–0.005 μg/day.^108^ Taneepanichskul et al. (2006) —estimated median infant exposure: Month 1: 19.86 ng/kg/day; Month 2: 15.08 ng/kg/day; Month 4: 10.45 ng/kg/day.^109^ Braga et al. (2015) —milk *C*_max_: 0.03 ng/mL; *T*_max_: ~2 weeks; milk‐to‐plasma ratio: 0.07; estimated infant exposure: 0.003 μg/day.^110^
Depot medroxyprogesterone acetate (DMPA) *IM or SC injection* *Every 3 months*	Injectable 150 mg (IM) 104 mg (SC)	No direct breastmilk pharmacokinetic data
Segesterone acetate + ethinyl estradiol *Vaginal* *1 year (cyclic use)*	Ring (103 mg + 17.4 mg)	No direct breastmilk pharmacokinetic data
Norethisterone enanthate *IM injection* *Every 2 months*	Injectable (200 mg)	No direct breastmilk pharmacokinetic data

*C*_max_, maximum drug concentration; *C*_ss_, steady‐state drug concentration; IM, intramuscular; IUD, intrauterine devices; SC, subcutaneous.

According to the Centers for Disease Control and Prevention (CDC) in its 2024 U.S. Medical Eligibility Criteria for Contraceptive Use,^64^ progestin‐only LARCs, such as the etonogestrel implant and levonorgestrel‐releasing IUDs are classified as Category 1 or 2 for breastfeeding individuals, indicating no restriction or advantages outweighing risks. The American College of Obstetricians and Gynecologists (ACOG) also supports immediate postpartum initiation of LARCs, noting their lack of negative impact on milk production or infant growth, and emphasizing their role in reducing unintended pregnancies.^65^ The World Health Organization (WHO) similarly recognizes progestin‐only methods as compatible with breastfeeding, and encourages their use to support maternal and child health goals.^66^ Collectively, these guidelines affirm that LARCs are a clinically appropriate and evidence‐based choice for postpartum contraception in lactating individuals.

#### What it means

Taken together, these reflect a growing commitment to understanding drug safety during lactation across diverse therapeutic areas. A few considerations are presented below:

- Exposure metrics: The milk‐to‐plasma area under the curve (AUC) ratio is considered the most reliable estimate of how much a drug enters breastmilk across the dosing interval.^52^ The infant dose is estimated using milk concentration multiplied by the volume of breastmilk an infant consumes daily.^67^ Relative infant dose expresses the resulting infant weight‐adjusted oral dose as a percentage of the maternal weight‐adjusted dose. Getting sufficient data to derive the AUC will be challenging for long‐acting therapeutics. Use of daily oral infant dose could be misleading for long‐acting drugs with no daily oral adult formulation (e.g., LEN). Therefore, quantification of drug levels in infant blood should be prioritized as it provides an absolute estimate of systemic exposure.
- Exposure over time: Long‐acting formulations maintain therapeutic drug levels in maternal plasma for weeks to months. This sustained release can lead to ongoing low‐level drug transfer into breastmilk, potentially resulting in considerable infant exposure for some time after maternal dosing has ceased. Hence, an extended sampling duration will be needed in clinical lactation studies of long‐acting therapeutics.
- Delayed clearance in neonates: Some drugs have long half‐lives, and infants (especially neonates) have immature hepatic and renal systems, which may delay drug clearance. This can amplify the duration and intensity of exposure, particularly for lipophilic drugs that accumulate in fat or have enterohepatic recirculation.
- Potential for adverse effects: Although most available data suggest low RID for many long‐acting agents, the risk of adverse effects cannot be ruled out—especially for drugs with central nervous system activity (e.g., antipsychotics). Sedation, EPS, or neurodevelopmental effects may occur with prolonged exposure, even at low levels, depending on the drug's mechanism of action and infant sensitivity.
- Challenges in monitoring and attribution: Passive and prolonged exposure through lactation makes it difficult to monitor infant drug levels and to confidently attribute clinical symptoms to specific drug exposures. This poses significant challenges for pharmacovigilance, potentially delaying the identification of adverse drug effects. Existing lactation safety classifications may not adequately reflect the pharmacokinetics of long‐acting formulations and should be updated accordingly. To better capture delayed or late‐onset outcomes that may emerge beyond the immediate postpartum period, long‐term follow‐up studies leveraging real‐world data are essential. Accurate documentation of lactation history, maternal medication history, and relevant outcomes in these data sources will enhance signal detection and safety evaluation.
- Breastfeeding guidance: Current guidelines often recommend caution or avoidance of breastfeeding when data are lacking. However, blanket avoidance may not be justified if RID is low and maternal therapy is essential. Instead, individualized risk–benefit assessments are needed, incorporating maternal health, drug properties, infant age, and available pharmacokinetic data.
- Need for dedicated clinical lactation studies: Most long‐acting drugs lack dedicated lactation pharmacokinetic studies, and extrapolation from oral formulations may be misleading. Studies like DOLPHIN3 and LAMP are critical to fill this gap, enabling evidence‐based recommendations for postpartum drug use. An ongoing Phase 4 trial (NCT06346288) evaluating the efficacy of risankizumab for inflammatory bowel disease is assessing breastmilk concentrations of this immunomodulator in lactating women. Risankizumab is a monoclonal antibody targeting interleukin‐23 (IL‐23) and used primarily for moderate‐to‐severe plaque psoriasis and other inflammatory conditions. It is administered once every 12 weeks via subcutaneous injection.

## LA THERAPEUTIC TECHNOLOGIES AND INNOVATIONS FOR MATERNAL HEALTH PRIORITIES

LA DDS alter the pharmacokinetics of drugs by modifying their absorption, distribution, metabolism, or excretion profiles to achieve sustained therapeutic levels over extended periods. We recently reviewed the various technologies that enable LA drug delivery, focusing on applications in infection therapeutics.^21^ We previously proposed a tiered approach to studying long‐acting therapeutics during pregnancy and lactation.^68^ It incorporates formulation risk definition based on LA formulations categorization by the FDA hinged on the predictability of drug release rate and the risk of clinically relevant biodistribution changes compared with the free drug.

LA DDS are broadly categorized into injectables—including carrier‐enabled (e.g., liposomes, polymeric nanoparticles) and noncarrier‐enabled formulations—and noninjectables, such as implantable (e.g., subdermal rods) and nonimplantable systems (e.g., transdermal patches).^21^ To optimize therapeutic performance, the drug payload is often pre‐modified to enhance physicochemical properties, improve stability, and tailor pharmacokinetics. Strategies such as prodrug derivatization, particle size reduction, and encapsulation in lipid or polymeric carriers are commonly employed to extend systemic circulation, control release rates, and improve biodistribution—ultimately enabling sustained therapeutic effects with reduced dosing frequency. Where nanomaterials are involved—whether as modified drugs, carriers, or excipients—their biological fate and safety profile must be rigorously assessed. Nanoparticles possess an enhanced ability to cross sensitive biological barriers such as the blood–brain barrier and the placenta, raising concerns about unintended fetal exposure.^69^ Nonbiodegradable components, in particular, pose risks due to their potential to accumulate and persist in biological tissues, which may lead to adverse effects from chronic exposure.^70^ In maternal health contexts, it is essential to rule out unintended accumulation in both maternal and fetal compartments through Developmental and Reproductive Toxicology studies. These assessments are critical for ensuring safety during pregnancy and lactation.

As highlighted in previous sections, several LA DDS have already been applied in maternal health settings, including during pregnancy and lactation. These technologies will likely continue to feature in upcoming LA therapeutics as they are supported by a growing body of clinical data and have not raised major safety concerns to date.

### Injectable noncarrier systems

Technologies in this group include oil depots, aqueous drug particle suspensions, and in situ forming implant (ISFI) or gel. The absence of a carrier system allows for a relatively higher loading of the active pharmaceutical ingredients compared with career‐enabled systems. Depending on the Biopharmaceutics Classification System class of the drug, multiple excipients are often used to enhance formulation properties (**Table** **4**). For instance, Class II drugs (low solubility, high permeability) may need solubilizers or particle size reduction, whereas Class IV drugs (low solubility, low permeability) often require more complex excipient strategies. Excipients used in LAI formulations must meet strict safety standards; hence, the use of those with established safety profiles and minimal systemic exposure. The FDA maintains a database of Generally Recognized As Safe (GRAS) substances, including excipients. This includes safety designations 1 to 5, where 1 denotes no evidence of hazard, and 2–5 indicates increasing levels of uncertainty or insufficient data. A generic LAI is expected to satisfy regulatory Q1 and Q2 sameness requirement, that is, contain the same inactive ingredients (Q1) and in the same concentration (Q2) as the reference listed drug. Since LAI are necessarily associated with prolonged exposure, it is reasonable to expect that the same requirement for excipients intended for long‐term clinical exposure will apply as per IPEC^71^ and FDA guidance.^72^

**Table 4:** Examples of long‐acting drug delivery systems used for maternal health indications

Technology	Description of key features	Common excipients	Example use cases for maternal health indications
Injectable noncarrier systems			
Oil depot	Characterized by slow dissolution from the injection site. Provides sustained drug release over weeks or months. Ideal for lipophilic drugs.	Sesame oil, castor oil, and peanut oil—primary vehicles for drug dissolution and depot formation. Benzyl benzoate, benzyl alcohol—co‐solvents to adjust viscosity and solubility. Surfactants (e.g., polysorbate 80) —to stabilize emulsions or suspensions. Phospholipids—used in lipid‐based depots for biocompatibility and controlled release.	Pregnancy: Fluphenazine decanoate (25 mg in 1 mL; SC/IM). It may be used if the benefits outweigh the risks. Third‐trimester exposure may cause neonatal withdrawal symptoms.
Aqueous drug particle suspensions	Enable controlled release of poorly soluble drugs; suitable for long‐acting injectables.	Suspending agents: for example, carboxymethylcellulose, methylcellulose and polysorbates are used to maintain uniform dispersion. Buffers: for example, phosphate buffer and citrate buffer are used to maintain pH stability. Preservatives: for example, benzyl alcohol and phenol are used to prevent microbial growth. Stabilizers: for example, EDTA and sorbitol are used to prevent aggregation or degradation.	Pregnancy: Cabotegravir (900 mg in 3 mL SC) and rilpivirine (600 mg in 3 mL SC) used once month or every two months SC injection for HIV treatment. Several reports of pregnancy exposure with no safety signal. Lactation: Depot medroxyprogesterone acetate (DMPA) is primarily used for contraception and known to be safe for use immediately postpartum in breastfeeding women. It is available as DMPA‐IM or DMPA‐SC.
In situ forming gels and implants.	Forms a solid depot under the skin upon contact with body fluids. Gradually releases payload over an extended period via diffusion and biodegradation.	Biodegradable polymers: for example, PLGA (poly(lactic‐co‐glycolic acid)), PLA, and PCL form the matrix for sustained release. Organic solvents: for example, N‐methyl‐2‐pyrrolidone (NMP), dimethyl sulfoxide (DMSO), dissolve the polymer and drug, then diffuse out postinjection. Plasticizers or viscosity modifiers: for example, triacetin and PEGs are used to adjust injectability and release rate. Phospholipids: sometimes used to enhance depot formation and biocompatibility.	Pregnancy: extended‐release SC buprenorphine for maintenance and treatment of moderate to severe opioid use disorder. Considered safe during pregnancy. It reduces risk of neonatal abstinence syndrome severity. Lactation: considered compatible with breastfeeding; low levels in breast milk and minimal oral bioavailability in infants.
Non‐injectable implantable			
Polymeric implant	Characterized by slow release via diffusion, degradation, or erosion. Made from biodegradable or nonbiodegradable polymers.	Ethylene vinyl acetate copolymer: acts as a core matrix and outer skin of the implant. Nonbiodegradable polymeric structure sustains drug release over time, ensures mechanical stability, and biocompatibility. Barium sulfate (in some products): makes the implant radiopaque, allow for X‐ray visualization to aid implant localization if unpalpable.	Lactation: Etonogestrel subdermal implant is used as a long‐acting reversible contraceptive. Considered compatible with breastfeeding; low levels in breast milk and minimal oral bioavailability in infants.
Titanium implant	Drug‐eluting titanium‐based structures for localized, long‐acting delivery, and to reduce systemic side effects.	A biocompatible titanium core for structural support, combined with drug‐loaded polymeric (e.g., PLGA, chitosan, collagen) and/or ceramic coatings (e.g., hydroxyapatite, tricalcium phosphate). Porous or nanostructured surfaces facilitate osseointegration and controlled release. Optional microreservoirs or carriers used for sustained or targeted drug delivery.	Used in orthopedic and dental applications to combine mechanical support with therapeutics (antibiotics, anti‐inflammatory agents, or bone‐regenerative drugs). Pregnancy: titanium itself is not contraindicated during pregnancy; however, concerns arise from the implantation procedure, including imaging, anesthesia, potential material degradation, and postoperative medications. Elective implantation is typically deferred until postpartum unless clinically indicated. Lactation: no direct evidence is available to guide use during lactation. While titanium is generally stable, rare degradation could lead to elevated systemic and breastmilk levels, posing a potential risk.
Osmotic pump implant	Uses osmotic pressure to deliver drugs at a constant rate over time.	No excipients. Uses a smartphone app to control drug release (e.g., an insulin pump and a continuous glucose monitor).	Hybrid closed‐loop automated insulin delivery system for diabetes control. Automatically adjusts insulin delivery based on real‐time glucose readings and helps maintain tight glycemic control. Pregnancy and lactation: shown to be safe and effective during pregnancy and lactation, with improved outcomes.

IM, intramuscular; SC, subcutaneous.

Importantly, GRAS designation does not require reproductive toxicity testing; hence, there is a general lack of pregnancy‐specific safety information on GRAS excipients. Further considerations for pregnancy‐safe LAI formulations include the following: (i) avoid excipients with teratogenic or embryotoxic potential; (ii) prioritize excipients that have been used safely during pregnancy for parenteral formulations; (iii) conduct preclinical reproductive toxicity studies for novel excipients; and (iv) monitor long‐term maternal and fetal outcomes in pregnant populations.

### Noninjectable implantable systems

Polymeric, electronic, titanium, and osmotic pump implants represent a class of LA DDS offering unique advantages, including localized delivery, bypass of physiological barriers, programmable release, modular design, and integration with smart materials (**Table** **4**). A key benefit is retrievability, allowing removal or replacement in case of adverse effects or therapy discontinuation, enhancing safety and treatment flexibility. While no published examples currently exist, retrievable LA DDS could be especially valuable for women of childbearing age receiving therapies with uncertain or known gestational risks, offering a reversible option for reproductive planning—similar to the reversibility seen in LARCs.

Injectables often require skilled administration and infrastructure, limiting their use in low‐resource settings. Emerging technologies aim to combine implant retrievability with the convenience of self‐administered injectables. For example, a biodegradable ISFI retrievable up to 180 days maintained protective CAB plasma levels for 11–12 months, with an 11‐fold decline post‐removal.^73^, ^74^ Similarly, SLIM technology—a polymer‐sparing, self‐aggregating LA injectable—achieves sustained release via crystal compaction, enabling high drug loading and compatibility with small‐gauge needles (25 G) for self‐administration.^75^ Key attributes making these technologies suitable for pregnancy and lactation include biodegradability, retrievability, and low polymer‐to‐drug ratios.

### Flip‐flop kinetics and fetal/infant drug exposure

**Figure 1:**
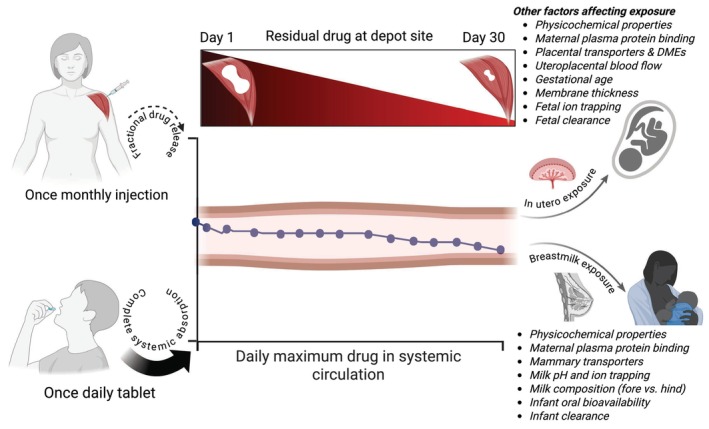
Flip‐flop kinetics of long‐acting therapeutics and in utero fetal and breastfed infant drug exposure. Long‐acting formulations like depot injections and implants are usually designed to achieve systemic drug levels comparable to daily oral dosing. Fetal exposure in utero and infant exposure through breastfeeding largely depend on the extent of drug absorption into maternal systemic circulation from the depot site. In the absence of formulation‐induced changes in biodistribution, these exposures are expected to be equivalent to those observed with daily oral administration. Other factors also play significant roles, including those listed in this image. Abbreviations: DMEs, drug‐metabolizing enzymes.

In the context of maternal therapeutics, in utero and breastmilk drug exposure are determined by the drug concentration in the systemic circulation (**Figure** **1**). For the LA DDS described above—injectable noncarrier systems and non‐injectable implantable platforms—only a fraction of the administered drug reaches systemic circulation due to ‘flip‐flop kinetics’. These systems are typically engineered to maintain plasma concentrations comparable to daily oral formulations, while minimizing peak‐trough fluctuations and enhancing pharmacokinetic stability.

Flip‐flop kinetics refers to a situation where a drug's absorption rate is slower than its elimination rate; this is true of LAI formulations. Thus, the absorption rate—rather than the elimination rate—becomes the rate‐limiting step in determining the drug's plasma concentration over time. Hence, the terminal phase of the concentration‐time curve in long‐acting formulations reflects absorption, not elimination. In contrast, a daily orally administered drug is absorbed quickly and eliminated more slowly, and the terminal phase of the concentration‐time curve reflects elimination.

### Time to steady‐state and long pharmacokinetic tail after discontinuation

Therapeutics delivered via LA DDS—such as injectable depots and ISFI—often require extended periods to reach steady‐state concentrations. This delay is attributed to residual drug at the administration site, which stabilizes slowly even after multiple doses. The clinical relevance is heightened during pregnancy, where physiological changes (e.g., increased plasma volume, altered protein binding, enhanced hepatic metabolism) accelerate drug clearance and reduce systemic exposure.^76^ Achieving a steady‐state prior to conception may help mitigate these fluctuations, ensuring therapeutic efficacy in conditions like HIV prevention or psychiatric care.

A hallmark of LA DDS is the prolonged pharmacokinetic tail following discontinuation, driven by slow depot dissolution and tissue redistribution. While beneficial for adherence, this extended tail complicates management during pregnancy and lactation, where rapid cessation may be necessary due to emerging safety concerns. In a CAB (nanosuspension) HIV PrEP trial, plasma levels remained detectable for up to 67.3 weeks post‐discontinuation (IQR: 29.1–89.6; range: 17.7–225.5).^77^ For the retrievable ISFI, plasma concentrations declined 11‐fold after removal but persisted for months, with multiphase elimination rather than immediate clearance.^73^, ^74^

This persistence poses challenges for pregnancy planning and lactation safety, as fetal and neonatal drug exposure may continue despite treatment cessation. PBPK modeling of CAB and RPV confirmed ongoing exposure during breastfeeding even after discontinuation in early pregnancy.^78^ Understanding and predicting tail‐phase kinetics is essential for designing bridging strategies (e.g., oral drug coverage) to prevent resistance or toxicity. Importantly, risk mitigation strategies must account for long‐term exposure beyond active treatment.

### Pharmacokinetics modeling in study design, exposure characterization, and dose optimization

Pharmacokinetic models use mathematical equations to describe and predict drug disposition in biological systems.^79^ Among the most widely used are popPK and PBPK models.^80^ These computational tools are particularly valuable in addressing logistical and ethical challenges associated with studying long‐acting therapeutics during pregnancy and lactation. Moreover, pharmacokinetic modeling enhances the utility of limited pharmacological data, enabling more informed decision‐making in data‐scarce settings.^76^, ^81^

PopPK models rely heavily on clinical data to build base models and conduct covariate analyses. They can incorporate sparse pharmacokinetic data pooled from multiple individuals, thereby reducing the number of samples required per participant and minimizing associated logistical and ethical burdens during study design.^81^, ^82^ In contrast, PBPK models integrate drug‐specific and system‐specific parameters to simulate drug‐body interactions and predict drug disposition.^83^ Once validated in one population (e.g., nonpregnant adults), PBPK models can be adapted to others (e.g., pregnant or lactating women), even with limited clinical data. However, successful adaptation requires prior knowledge of and adjustments for physiological differences between populations to ensure accurate in silico predictions.^82^, ^83^ PBPK models developed for oral drugs can also be adapted for long‐acting therapeutics by accounting for differences in formulation and route of administration.^84^ This adaptability makes PBPK modeling especially useful for predicting drug behavior in pregnant and breastfeeding populations, where clinical PK data are often scarce. Nonetheless, the availability of pharmacokinetic data in the target population remains important for validating model predictions and increasing confidence in their performance.

PBPK models can assess pregnancy‐induced changes in drug exposure and predict fetal and breastfed infant exposure, informing safe use of long‐acting therapeutics during pregnancy and lactation.^76^, ^85^, ^86^ We used a pregnancy PBPK model to predict fetal exposure to LA‐CAB/RPV at therapy initiation, reporting median cord‐to‐maternal blood ratios of 1.71 and 0.88, respectively, at delivery. Monthly RPV *C*_trough_ was below the clinical effective concentration (50 ng/mL) at week 12 in 40% of the virtual pregnant cohort, while CAB *C*_trough_ remained above 664 ng/mL throughout pregnancy.^76^ During therapeutic drug monitoring for pregnant women on LA‐RPV, pregnancy may affect total and unbound RPV concentrations differently. Virologic suppression may still occur despite reduced total exposure if unbound levels are adequate.^87^ Achieving a steady‐state before conception may mitigate pregnancy‐related changes. PBPK models have also simulated tail‐phase pharmacokinetics of LA‐CAB/RPV when discontinued during pregnancy, including fetal exposure.^78^ Similar models could assess continued use or estimate oral PrEP coverage duration needed post‐discontinuation to prevent resistance. When multiple dosing strategies exist, PBPK modeling can compare fetal exposure across regimens, guiding selection to minimize fetal drug exposure. Comparisons between long‐acting and oral formulations are also possible. Pregnancy PBPK models predicted cumulative monthly fetal exposures over 130% and 160% higher with long‐acting vs. oral olanzapine and aripiprazole, respectively.^86^ PBPK models can be adapted to simulate infant exposure during lactation by reversing pregnancy‐induced physiological changes.^85^, ^88^ This enables formulation comparisons to assess infant drug exposure and inform safety for breastfeeding women using long‐acting drugs.

Beyond exposure prediction, pharmacokinetic modeling plays a critical role in clinical trial design. PBPK models can simulate various dosing strategies and clinical scenarios to optimize study design for long‐acting therapeutics in pregnant and lactating populations. These models can be integrated into a popPK framework to simulate clinical trials at the population level.^82^ For example, model‐based simulations can estimate the proportion of individuals likely to fall above or below therapeutic concentration thresholds under different dosing regimens.^76^ They can also support risk assessments by predicting how sample size and study design influence the likelihood of observing outliers, thereby informing data safety monitoring boards and guiding real‐time decision‐making during trials.

Conducting intensive pharmacokinetic sampling in pregnant and lactating women is often challenging. However, sparse pharmacokinetic data—including from therapeutic drug monitoring—can be pooled for popPK analysis. Since dosing of long‐acting formulations is often directly observed, sparse data do not suffer from limitations like uncertainties about adherence often experienced with daily oral medications. It is particularly useful when wide interindividual variability exists. In such cases, popPK models can identify covariates that influence drug disposition and support individualized dosing strategies to optimize efficacy and minimize adverse outcomes.^89^ When sufficient data are available, popPK analyses can be tailored specifically to pregnant and lactating women to address their unique pharmacological needs.^89^

PK modeling is also invaluable for dose optimization. PBPK models can predict long‐acting drug doses that achieve equivalent plasma concentrations during pregnancy and lactation.^76^, ^84^ Additionally, PBPK modeling has been used to study DDIs—for example, between rifampicin and LA‐CAB/RPV in adults.^90^ A pregnancy PBPK model has also been developed to evaluate DDIs between ritonavir‐boosted atazanavir and rifampicin.^91^ Combining these approaches could enable the development of pregnancy‐specific PBPK models to assess DDIs involving long‐acting therapeutics and co‐medications, supporting personalized dosing strategies for pregnant women. Mechanistic insights from PBPK models are also critical for calibrating popPK models, which are more readily used for individualized dosing.

However, both modeling approaches face limitations. A key challenge for popPK analysis in pregnancy and lactation is the limited availability of data. Conversely, PBPK models often rely on simplified absorption models—such as first‐order equations with fitted release rates—to describe drug release from intramuscular depots. While useful, these models may not adequately capture the influence of factors like body mass index, which has been shown to affect absorption from long‐acting formulations.^76^, ^92^ Despite growing interest, the application of PBPK modeling to long‐acting therapeutics in maternal populations remains limited. Over the past 25 years, approximately 125 pregnancy and 34 lactation PBPK models have been published, yet only three pregnancy PBPK models have focused on long‐acting therapeutics, and none has been published for lactation.

## CONCLUSIONS

Successful development, adoption, and implementation of long‐acting therapeutics that will address maternal health priorities will likely be influenced by several critical early and concurrent actions. This is best illustrated with the example of a therapy for PPH, the largest direct cause of maternal death worldwide. About 70% of PPH deaths occur after delivery, primarily due to uterine atony—insufficient uterine contraction following childbirth. Oxytocin, the current standard for PPH prevention, has limited real‐world effectiveness in low‐resource settings due to its heat sensitivity and dependence on cold‐chain storage, which compromises drug potency and quality.

To address this critical gap, a multi‐stakeholder partnership was formed involving the Program for Appropriate Technology in Health (PATH), Merck for Mothers, and the Kenya Ministry of Health. This collaboration led to the identification, development, and evaluation of heat‐stable carbetocin (HSC), a long‐acting oxytocin analogue that does not require refrigeration and retains efficacy in high‐temperature environments.^93^ The strategy began with a global technology assessment (2012–2014) led by PATH and Merck for Mothers, which screened over 30 maternal health innovations. HSC emerged as a promising candidate due to its pharmacological profile and suitability for low‐resource settings. The CHAMPION trial, coordinated by the WHO and supported by Merck and Ferring Pharmaceuticals, enrolled nearly 30,000 women across 10 countries, including Kenya. Results demonstrated that HSC was non‐inferior to oxytocin in preventing blood loss ≥500 mL, validating its potential as a climate‐resilient alternative for PPH prevention.^94^ Following the trial, HSC was added to the WHO Model List of Essential Medicines in 2019 for use in settings where oxytocin quality cannot be guaranteed. Kenya subsequently procured the continent's largest batch of HSC and integrated it into public sector maternal health programs, supported by policy shifts and provider training. This initiative exemplifies how public‐private partnerships can accelerate access to life‐saving interventions and reduce maternal mortality in resource‐constrained environments.

Both LEN and LA‐CAB/RPV are now being advanced through strategic public‐private partnerships aimed at improving access in low‐resource settings, particularly for maternal populations. LEN's rollout involves Gilead, PEPFAR, and the Global Fund, with commitments to non‐profit supply, voluntary licensing to generic manufacturers, and prioritization of pregnant and breastfeeding women. Progress for LA‐CAB/RPV is more complex: while ViiV Healthcare has licensed CAB to the Medicines Patent Pool to enable generic production, the RPV component currently relies on Janssen's direct supply commitments and bilateral agreements, pending broader patent expirations. The LEN and LA‐CAB strategies mirror the approach used for HSC, where a multi‐stakeholder collaboration led to the development and deployment of a climate‐resilient uterotonic for postpartum hemorrhage. In all cases, the focus is on formulation innovation, equitable access, and implementation science to address critical gaps in maternal health care. In contrast, LARCs face slower innovation and implementation due to a lack of global coordination, with access constrained by provider training gaps, social stigma, and concerns about coercion.^95^, ^96^ Similarly, LAI antipsychotics deployment in LMICs is limited by cost, infrastructure, and policy gaps. Broader investment and policy alignment are needed to match the impact of HSC, LEN, and LA‐CAB/RPV.

The success stories illustrate how coordinated public‐private partnerships, global clinical trials (that include pregnant and postpartum women), and strategic access mechanisms such as voluntary licensing and no‐profit supply agreements, can enable rapid scale‐up of interventions for maternal health priorities, including where they matter the most. As long‐acting therapeutics become mainstream strategies for treatment and prevention, it is imperative that maternal health priorities are fully integrated—and that women who are pregnant or breastfeeding are deliberately included—in research, policy development, and access planning.

## FUNDING

This work was supported by Unitaid (grant number 2020‐38‐LONGEVITY). AO receives funding from the Wellcome Trust (227288/Z/23/Z) and United States NIH (through LEAP award number 2R24AI118397–11). EDW received funding from the United States NIH through a mentored career development award (number K23AI150349). CW was funded by the United Kingdom National Institute for Health and Care Research (NIHR304266). The views expressed are those of the authors and not necessarily those of the funders. For open access, the authors have applied a CC‐BY public copyright license to any Author Accepted Manuscript version arising from this submission.

## CONFLICT OF INTEREST

Rachel Scott has participated in Advisory Board Meetings for ViiV Healthcare and Gilead Sciences. She has previously received research funding from both companies; all funding was managed by MedStar Health Research Institution. All other authors declared no competing interests for this work.
